# Supplementary material for: Nanoscale kinetics of asymmetrical corrosion in core-shell nanoparticles
Source: Nat Commun. 2018 Mar 8;9:1011. doi: 10.1038/s41467-018-03372-z (PMC5843659; doi:10.1038/s41467-018-03372-z)
Supplement: Supplementary file 1 — Supplementary Information [file 41467_2018_3372_MOESM1_ESM.pdf]

Description of Supplementary
File Name: Supplementary Information
Description: Supplementary Figures, Supplementary Movies, Supplementary Notes and Supplementary References

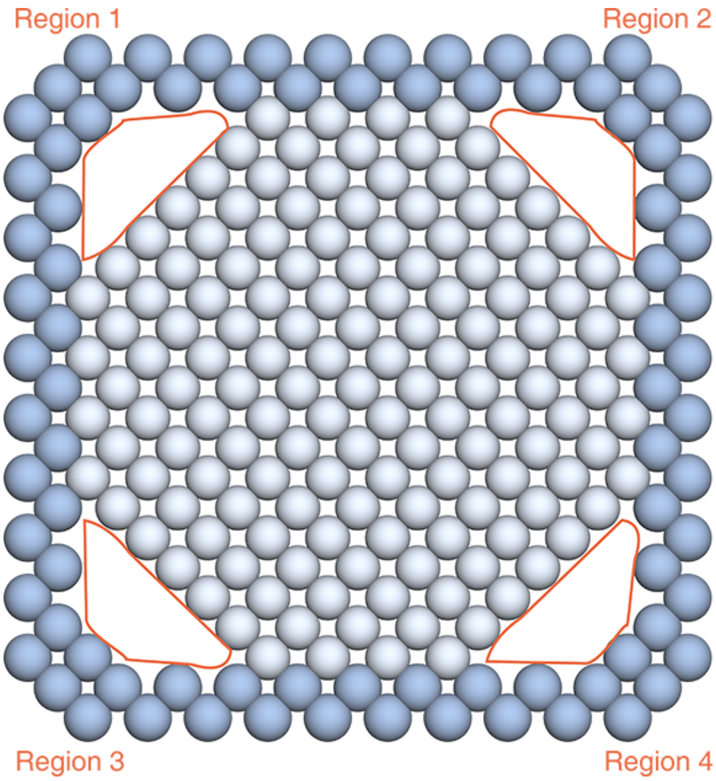

Supplementary Figure 1 | Illustration of the calculation of area. The value that total etched area along the same direction divided by the whole area of cube was defined as etching area (C_a).

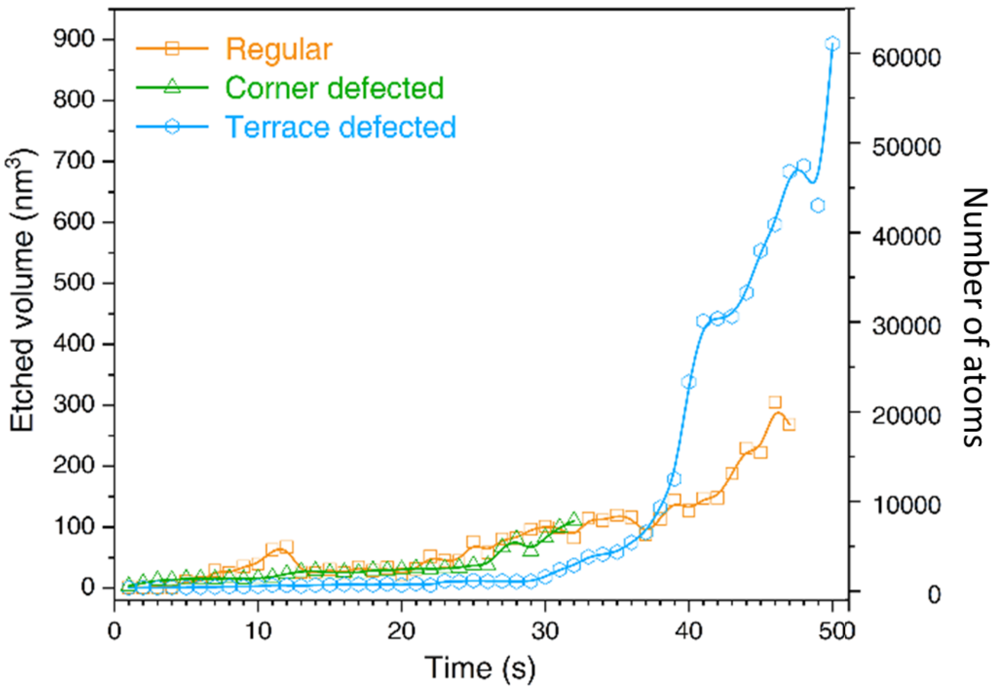

Supplementary Figure 2 | Volume and number of etched atoms. Curves of etched volume and etched atoms number as a function of time.

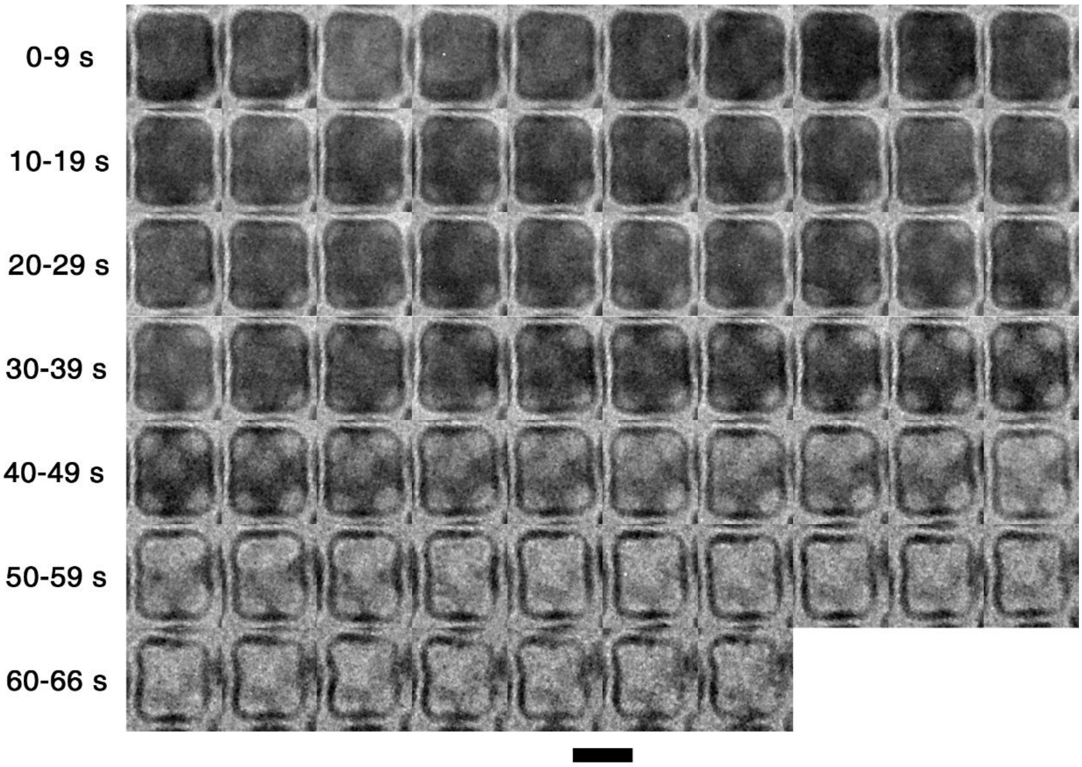

Supplementary Figure 3 | Etching process of regular cube. Time sequential TEM micrographs by second of etching process of internal Pd atoms in a single regular Pd@Pt cube. Scale bars in all panels are 10 nm. Electron beam current density used here is 68 pA/cm^2.

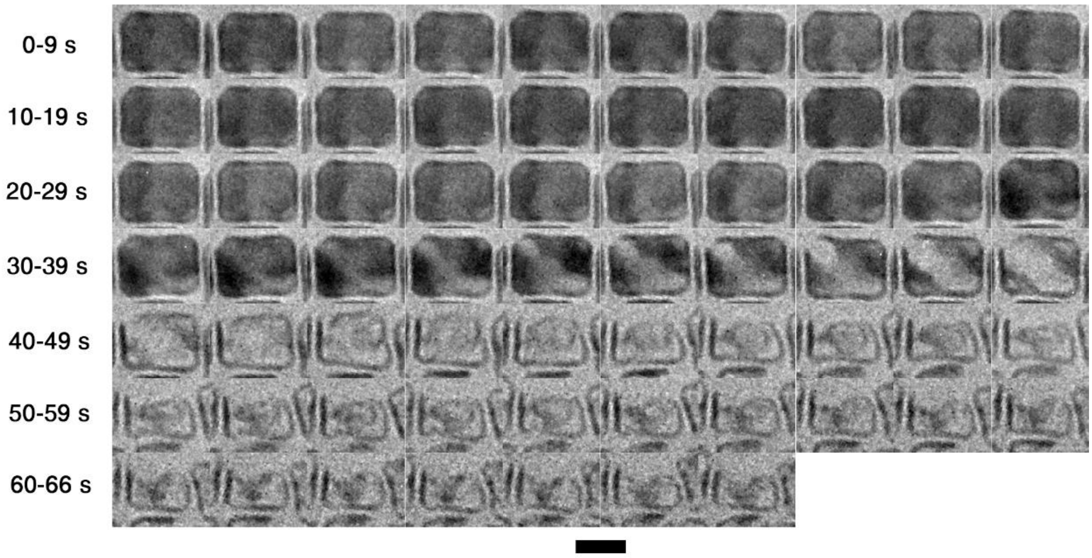

Supplementary Figure 4 | Etching process of corner defected cube. Time sequential TEM micrographs by second of etching process of internal Pd atoms in a single Pd@Pt cube with corner defect. Scale bars in all panels are 10 nm. Electron beam current density used here is 68 pA/cm^2.

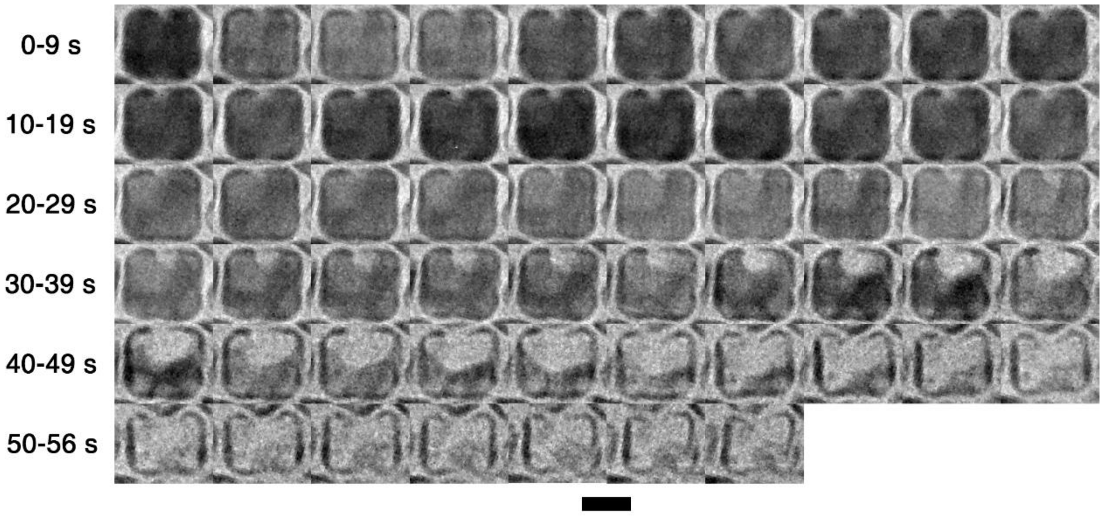

Supplementary Figure 5 | Etching process of terrace defected cube. Time sequential TEM micrographs by second of etching process of internal Pd atoms in a single Pd@Pt cube with terrace defect. Scale bars in all panels are 10 nm. Electron beam current density used here is 68 pA/cm^2.

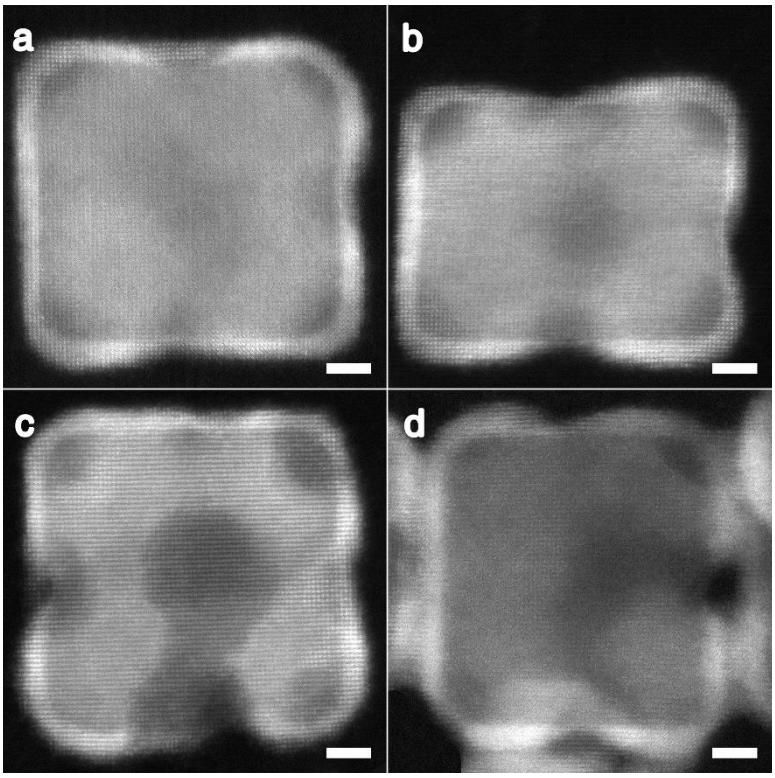

Supplementary Figure 6 | Different intermediate etching states. High-angle annular dark-field scanning transmission electron microscopy (HAADF-STEM) images of different intermediate states of etching of Pd@Pt cube with terrace defects. Scale bars in all panels are 2 nm.

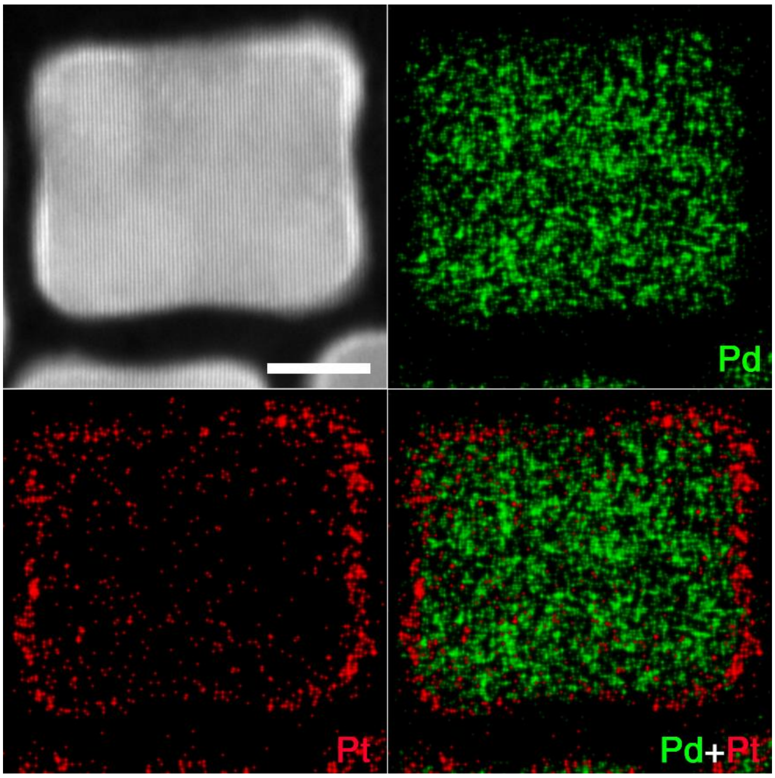

Supplementary Figure 7 | EDS elemental mapping of cube before etching. Energy dispersive spectra (EDS) elemental mapping of Pd@Pt cube before etching. Scale bar is 5 nm.

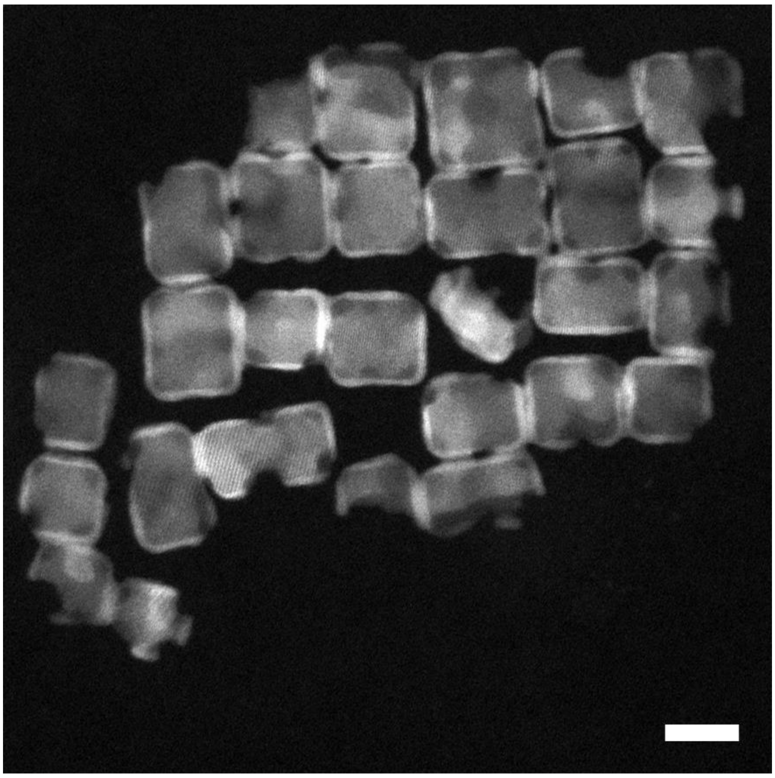

Supplementary Figure 8 | A large amount of Pt cages. Low magnification HAADF-STEM image of Pt cages after etching of Pd@Pt cubes. Scale bar is 10 nm.

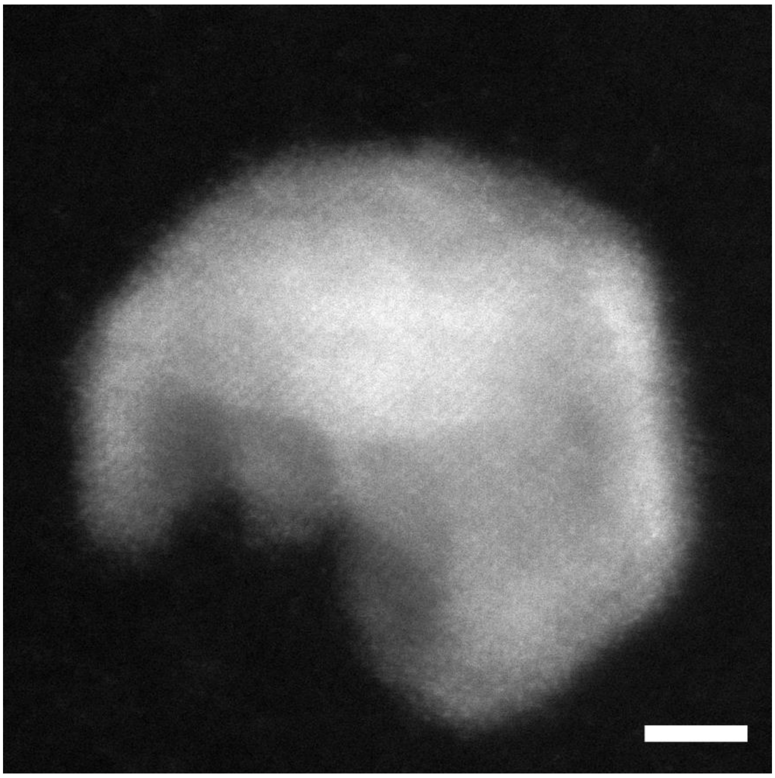

Supplementary Figure 9 | Collapse Pt cage. HAADF-STEM image of collapse Pt cage without the support from inner Pd cube after etching. Scale bar is 2 nm.

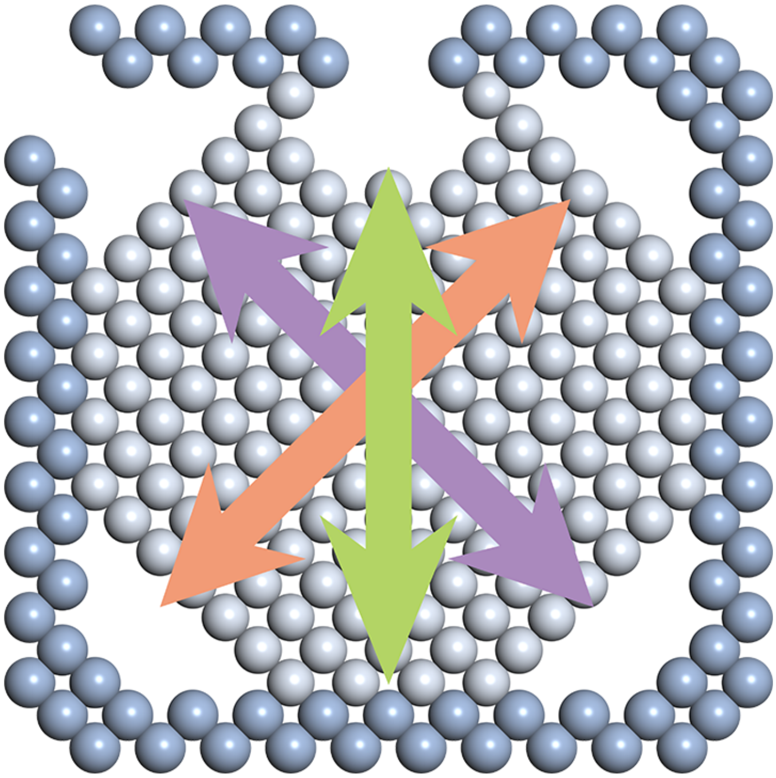

Supplementary Figure 10 | Illustration of the measured distance. The illustration of atomic structures demonstrates the measured distance of residual Pd atoms along directions.

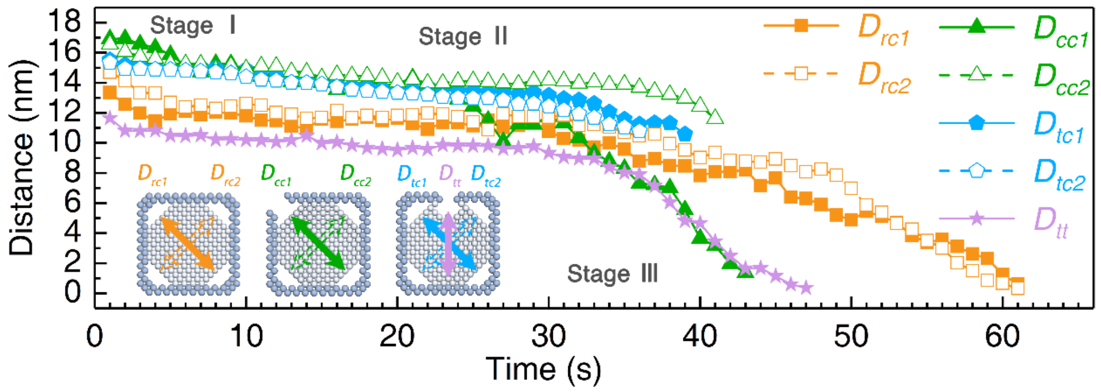

Supplementary Figure 11 | Measured distance of residual Pd atoms. Changes of the measured distance of residual Pd atoms to each direction as a function of time.

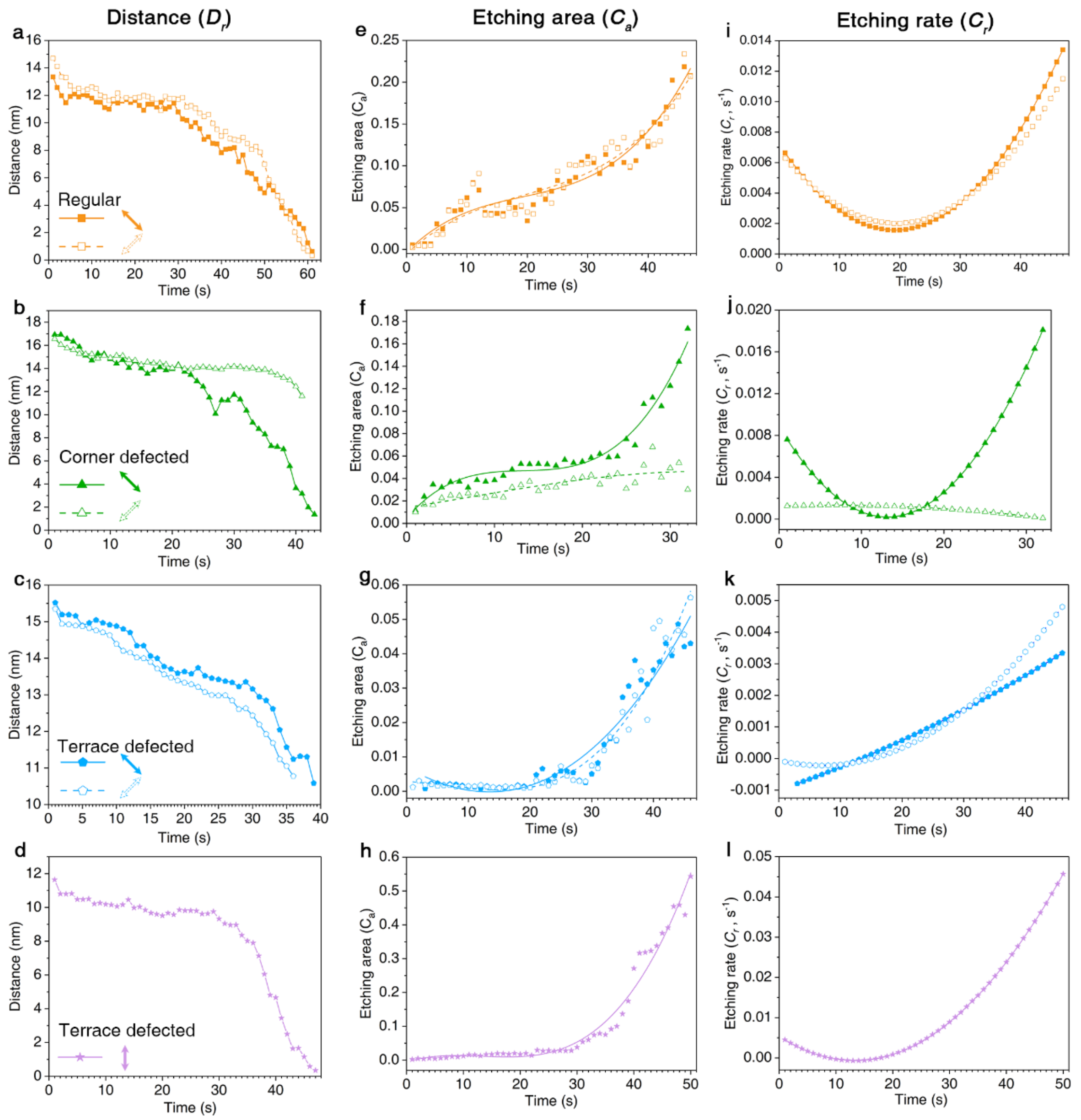

Supplementary Figure 12 | Comparison in three types cubes. Plots of measured distance, etching area and etching rate of regular, corner defected and terrace defected Pd@Pt cubes, respectively.

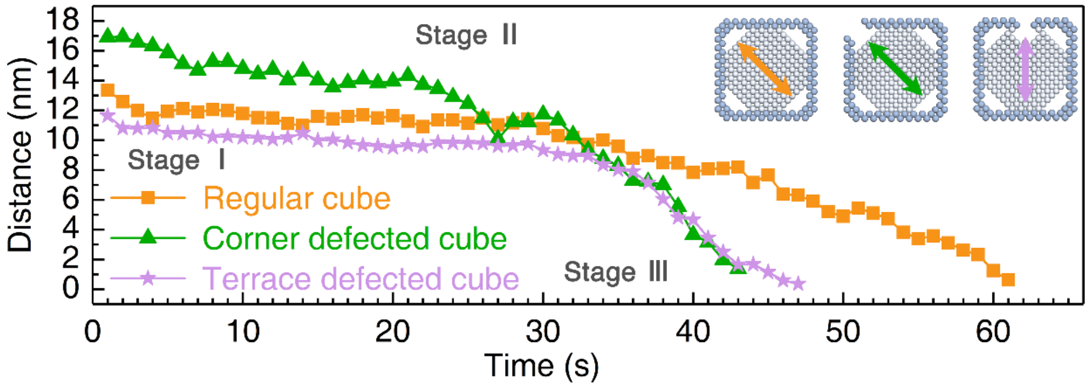

Supplementary Figure 13 | Measured distance of residual Pd atoms. Changes of the measured distance of residual Pd atoms to representative directions as a function of time in regular, corner and terrace defected cubes, respectively.

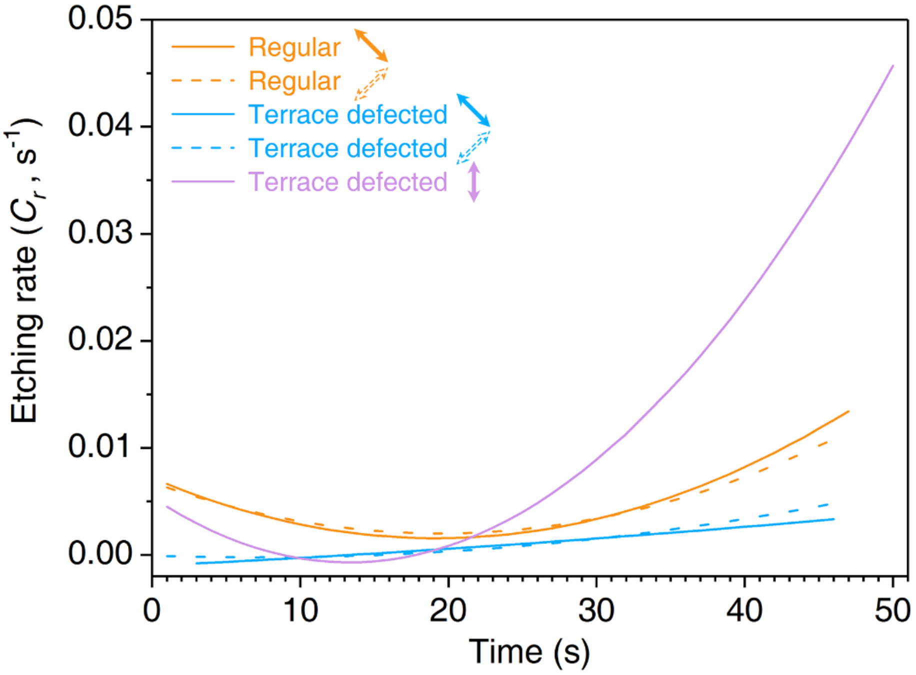

Supplementary Figure 14 | Comparison of etching modes. Etching modes comparison between halogen etching and galvanic etching.

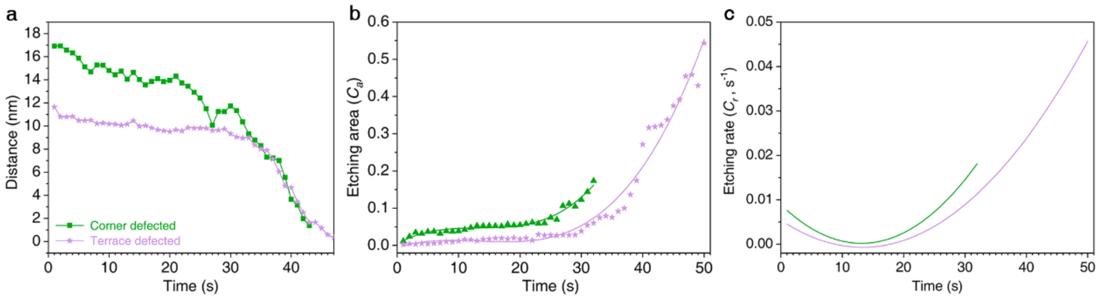

Supplementary Figure 15 | Comparison of halogen etching mode. Comparison of halogen etching mode between corner defected and terrace defected cubes. Distance (D_r) is measured before a random direction is etched completely. Data of area (C_a) are collected before two random holes are merged.

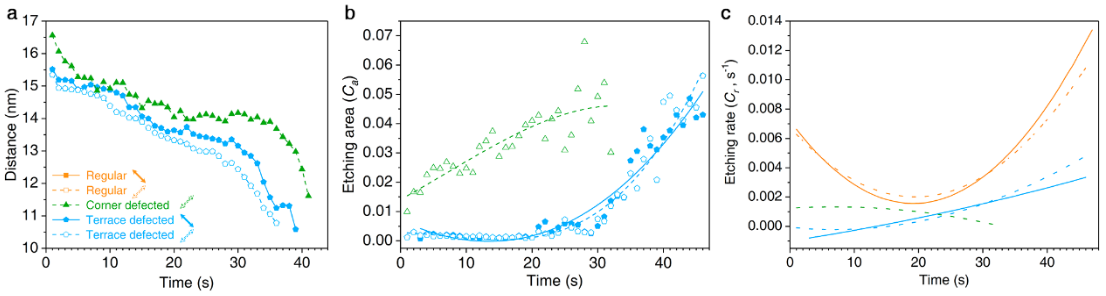

Supplementary Figure 16 | Comparison of galvanic etching mode. Comparison of galvanic etching mode between corner defected and terrace defected cubes.

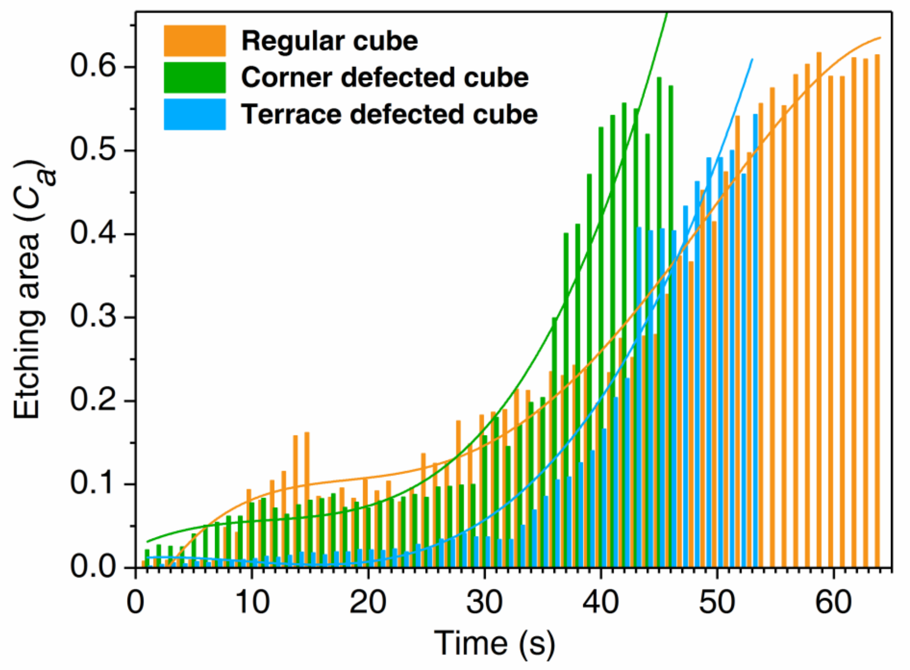

Supplementary Figure 17 | Comparison of entire etching area. Entire etching area ($\sum C_a$) in one cube as a function of time and the comparison between regular, corner defected and terrace defected cubes, respectively.

Supplementary Movies

Supplementary Movie 1. Movie showing the existence of bubbles in the liquid cell of in situ TEM holder. After the reaction solution was introduced into the liquid cell, bubbles were initially generated under electron beam irradiation, implying the existence of liquid.

Supplementary Movie 2. Movie showing the etching process of internal Pd atoms in a single regular Pd@Pt cube with the flow rate of 5 ul/min under the electron beam current density of 68 pA/cm^2.

Supplementary Movie 3. Movie showing the etching process of internal Pd atoms in a single corner defected Pd@Pt cube with the flow rate of 5 μl/min under the electron beam current density of 68 pA/cm^2.

Supplementary Movie 4. Movie showing the etching process of internal Pd atoms in a single terrace defected Pd@Pt cube with the flow rate of 5 μl/min under the electron beam current density of 68 pA/cm^2.

Supplementary Movie 5. Movie showing the etching process of internal Pd atoms in Pd@Pt cubes in large scale with the flow rate of 5 μl/min under the electron beam current density of 68 pA/cm^2.

Supplementary Table 1 | Average etching rate. The values of average of etching rate in corresponding period of time.

**Regular Pd@Pt cube**	**1-12 s**	**13-27 s**	**28-47 s**
	5.95×10^{-3} s^{-1}	2.67×10^{-3} s^{-1}	6.4×10^{-3} s^{-1}
**Corner defected Pd@Pt cube**	**1-5 s**	**6-23 s**	**24-32 s**
	6.31×10^{-3} s^{-1}	1.27×10^{-3} s^{-1}	1.42×10^{-2} s^{-1}
**Terrace defected Pd@Pt cube**	**1-29 s**		**30-50 s**
	9.35×10^{-4} s^{-1}		2.52×10^{-2} s^{-1}

Supplementary Note 1 | Comparison of etcing area and distance along different directions.

We also measured and plotted the distance of residual Pd (D_r) along each direction as a function of time (Supplementary Figure 11). The curves showed a similar trend (details of measurement can be found in Supplementary Figure 10). At beginning, etching initialized, Pd atoms at corners, which were high surface energy sites, were first etched easily leading to a fast etching rate (Supplementary Figure 12a-c), and gradually increased C_a (Supplementary Figure 12e-g). Subsequently, after Pd atoms at the corner were etched away, flat high-coordinated surfaces of {111} and {110} with relative lower energy appeared, and then etching slowed down, resulting in a plateau in ~11-30 s (Supplementary Figure 12a-c, e-g). Finally, inner Pd nanocrystal decreased a lot in size and formed as quasi-spheres with certain high index planes, which was highly active for etching. Therefore, etching rate became faster again due to a high index plane and the higher chemical potential from the small local surface curvature to highly curved regions based on Kelvin equation after 31 s (Supplementary Figure 12a-c, ~31-60 s)[1,2]. In the entire etching process, the etching rate C_r followed the pace of “high-low-high” (Supplementary Figure 12i-k), which was consistent with the “S” type of etching phenomenon as described above. However, etching along the direction of terrace defects only presented two stages, slow in the first 1-29 s on {100} facet with relative low surface energy, yet, fast from 30 s on the high index plane with higher surface energy (Supplementary Figure 12d, h, l). Selected three representative featured curves to show the stages in etching process, as shown in Supplementary Figure 13.

Supplementary Note 2 | Comparison of etching modes in cubes.

We further compared etching rate (C_r) of three types of Pd@Pt cubes along the featured direction to investigate the coherence between galvanic and halogen etchings. In the cases cubes with defects on corner and terrace, two etching modes (galvanic etching at the coating area and halogen etching at area close to defects) coexisted. C_r at the close to defects were much higher than C_r at the defects free area for most of the time, indicating that the halogen etching was faster than the galvanic etching. On the other hand, in Figure 1e, C_r of corner defected cube was lower than that of regular cube along UR-LL., This was because halogen etching close to the defects could largely consume the oxidative species, and suppress the galvanic etching at the Pt-capped corners. This phenomenon also occurred in the case of terrace defected cube, whose C_r from corners were all slower than those of cube without defects (Supplementary Figure 14). In the first 29 s, D_r for corner defected cube decreased with a loss of 50.6% (5.7 nm), however, D_r for terrace defected cube dropped with only a loss of 16.2% (1.9 nm) (Supplementary Figure 15a). The competition between the two etching modes can also be evidenced by the etching rate of area (C_a): the C_a of 0.11 for corner defected cube, in comparison with the C_a of 0.028 for cube with defect on terrace (Supplementary Figure 15b) in the first 29 s. In addition, in Supplementary Figure 15c, the C_r from defect on the corner was always higher than that from the cube with defect on the terrace. We can therefore attribute the difference in halogen etching to the two following points: 1) the higher chemical reactivity of corners resulted from their lower coordination number comparing with terraces[2]. 2) the high surface free energies in corner rather than in terrace[3]. Further comparison of galvanic etching between these two defected cubes can be seen in Supplementary Figure 16.

The galvanic etching in terrace defected cube was slower than that in corner defected cube at the region of 1-21s (Supplementary Figure 16c). At the region after 21 s when both halogen and galvanic etching are present in the process

of corrosion, the order of the rate of galvanic etching was regular cube > terrace defected cube > corner defected cube (Supplementary Figure 16c).

Supplementary References

1. Sambles, J., Skinner, L. & Lisgarten, N. An electron microscope study of evaporating small particles: the Kelvin equation for liquid lead and the mean surface energy of solid silver. *Proc. Math. Phys. Eng. Sci.* **318**, 507-522 (1970).

2. Jiang, Y. *et al.* In situ study of oxidative etching of palladium nanocrystals by liquid cell electron microscopy. *Nano Lett.* **14,** 3761-3765 (2014).

3. Xia, Y., Xia, X. & Peng, H.-C. Shape-controlled synthesis of colloidal metal nanocrystals: thermodynamic versus kinetic products. *J. Am. Chem. Soc.* **137,** 7947-7966 (2015).
