# Supplementary material for: Nanoscale kinetics of asymmetrical corrosion in core-shell nanoparticles
Source: Nat Commun. 2018 Mar 8;9:1011. doi: 10.1038/s41467-018-03372-z (PMC5843659; doi:10.1038/s41467-018-03372-z)
Supplement: Supplementary file 3 — Description of Additional Supplementary Files [file 41467_2018_3372_MOESM3_ESM.pdf]

Description of Additional Supplementary Files

File Name: Supplementary Movie 1
Description: Movie showing the existence of bubbles in the liquid cell of in situ TEM holder. After the reaction solution was introduced into the liquid cell, bubbles were initially generated under electron beam irradiation, implying the existence of liquid.

File Name: Supplementary Movie 2
Description: Movie showing the etching process of internal Pd atoms in a single regular Pd@Pt cube with the flow rate of 5 ul/min under the electron beam current density of 68 pA/cm^2.

File Name: Supplementary Movie 3
Description: Movie showing the etching process of internal Pd atoms in a single corner defected Pd@Pt cube with the flow rate of 5 µl/min under the electron beam current density of 68 pA/cm^2.

File Name: Supplementary Movie 4
Description: Movie showing the etching process of internal Pd atoms in a single terrace defected Pd@Pt cube with the flow rate of 5 µl/min under the electron beam current density of 68 pA/cm^2.

File Name: Supplementary Movie 5
Description: Movie showing the etching process of internal Pd atoms in Pd@Pt cubes in large scale with the flow rate of 5 µl/min under the electron beam current density of 68 pA/cm^2.
